# Phase Variation of Poly-N-Acetylglucosamine Expression in *Staphylococcus aureus*

**DOI:** 10.1371/journal.ppat.1004292

**Published:** 2014-07-31

**Authors:** Jamie L. Brooks, Kimberly K. Jefferson

**Affiliations:** Department of Microbiology and Immunology, Virginia Commonwealth University School of Medicine, Richmond, Virginia, United States of America; University of Tubingen, Germany

## Abstract

Polysaccharide intercellular adhesin (PIA), also known as poly-N-acetyl-β-(1–6)-glucosamine (PIA/PNAG) is an important component of *Staphylococcus aureus* biofilms and also contributes to resistance to phagocytosis. The proteins IcaA, IcaD, IcaB, and IcaC are encoded within the intercellular adhesin (*ica*) operon and synthesize PIA/PNAG. We discovered a mechanism of phase variation in PIA/PNAG expression that appears to involve slipped-strand mispairing. The process is reversible and RecA-independent, and involves the expansion and contraction of a simple tetranucleotide tandem repeat within *icaC*. Inactivation of IcaC results in a PIA/PNAG-negative phenotype. A PIA/PNAG-hyperproducing strain gained a fitness advantage in vitro following the *icaC* mutation and loss of PIA/PNAG production. The mutation was also detected in two clinical isolates, suggesting that under certain conditions, loss of PIA/PNAG production may be advantageous during infection. There was also a survival advantage for an *icaC*-negative strain harboring intact *icaADB* genes relative to an isogenic *icaADBC* deletion mutant. Together, these results suggest that inactivation of *icaC* is a mode of phase variation for PIA/PNAG expression, that high-level production of PIA/PNAG carries a fitness cost, and that *icaADB* may contribute to bacterial fitness, by an unknown mechanism, in the absence of an intact *icaC* gene and PIA/PNAG production.

## Author Summary

Staphylococcal polysaccharide intercellular adhesin (PIA), also known as β-1-6-linked N-acetylglucosamine (PNAG) plays a role in immune evasion and biofilm formation. Evidence suggests that under certain circumstances PIA/PNAG production is beneficial, whereas at times, it may be advantageous for the bacteria to turn production off. In *S. epidermidis*, PIA/PNAG can be switched off when an insertion sequence recombines into the intercellular adhesin locus (*ica*). In this study, we have found a short tandem repeat sequence in the *ica* locus of *S. aureus* that can undergo expansion and contraction. The addition or subtraction of non-multiples of three of this repeat shifts the reading frame of the *icaC* gene, resulting in the complete loss of PIA/PNAG production. We hypothesize that certain conditions that make the PIA/PNAG-negative phenotype advantageous during infection, such as the development of an effective immune response to PIA/PNAG on the bacterial surface, would select for repeat mutants. In support of this hypothesis, we found clinical isolates with expansion and deletion of the repeat. These findings reveal a new on-off switch for the expression of PIA/PNAG.

## Introduction

Phase variation functions as a reversible on/off switch for the expression of a particular gene. The result is commonly an alteration in the expression of some cell surface-expressed antigen. Slipped-strand mispairing is one mechanism that can lead to the production of a phase variant. Slipped-strand mispairing occurs during DNA replication when there is mispairing between mother and daughter DNA strands in regions of DNA that contain simple 1–10 nucleotide repeats [1]. This results in the addition or subtraction of one or more repeats that can bring about a change in transcriptional efficiency or shift the reading frame to alter or halt translation.

*Staphylococcus aureus* infections are responsible for an enormous loss of life; deaths from methicillin resistant *S. aureus* (MRSA) alone exceed 18,000 yearly in the United States, making it the leading cause of death by a single infectious agent [2]. Antibiotic resistance is a mounting problem and an effective vaccine is not yet available. Biofilm formation plays an important role, particularly in device-related infections, and it contributes to antibiotic failure and resistance of the bacteria to host immune defenses.

Biofilm formation is the aggregation of bacteria on a solid surface within a self-produced extracellular polymeric matrix. Formation of a biofilm confers several survival advantages to the resident bacteria. The biofilm provides protection from adverse environmental conditions such as heat, shear force, and UV damage; as well as protection from the host immune system and antibiotic challenge [3]. Biofilm bacteria are resistant to antibiotic levels up to 1,000-fold higher than planktonic bacteria that are genetically identical [4], [5]. A major component of the *S. aureus* biofilm extracellular matrix is the polysaccharide poly-N-acetylglucosamine.

The staphylococcal polysaccharide intracellular adhesin (PIA) is a high molecular weight polymer of β-1-6-linked N-acetyl-glucosamine (PNAG) [6], [7]. In addition to its role in intercellular adhesion and biofilm formation, PIA/PNAG also plays a role in immune evasion [8], [9]. Evidence suggests that antibodies against PIA/PNAG often recognize secreted PIA/PNAG rather than the surface-associated form, resulting in an ineffective immune response [8]. In contrast, an effective immune response against surface-associated PIA/PNAG, which can be directed by a conjugate vaccine, can successfully eradicate an infection [10]. Thus, PIA/PNAG protects the bacteria from immune defenses but under certain circumstances could actually be the target of an effective immune response.

PIA/PNAG is synthesized by the proteins encoded in the *icaADBC* intercellular adhesin locus [11], [12]. IcaA is a transmembrane glucosyltransferase that, together with IcaD, produces short PIA/PNAG oligomers [13]. IcaC is an integral membrane protein that is necessary for linking the short oligomers into longer polymer chains, and is thought to be involved in translocation of these chains to the cell surface [13]. Once there, IcaB is responsible for partial deacetylation of the PIA/PNAG molecule, which is required for retention at the cell surface [14].

A number of regulators modulate *icaADBC* transcription, including IcaR and CodY, which are repressors, and SarA and GraRS, which are positive regulators [15], [16], [17], [18]. Other regulatory mechanisms have been implicated as well and are described in a recent review [19]. We found previously that a 5-nucleotide deletion mutation within the *icaADBC* promoter was sufficient to induce constitutive transcription of the *icaADBC* genes and high-level PIA/PNAG production, resulting in a mucoid phenotype and strong biofilm production [15]. In the present study, we noted that growth of the mucoid strain in liquid culture resulted in the rapid accumulation of non-mucoid variants. We investigated this phenomenon and found that the most frequent mutation leading to the PIA/PNAG-off phenotype was a change in the number of a specific tandem repeat in *icaC*. This mutation was reversible and was found in clinical isolates as well. The PIA/PNAG-negative variants had a growth advantage over the PIA/PNAG-overproducing parent and rapidly predominated the cultures. This represents a newly recognized mechanism of PIA/PNAG regulation in *S. aureus*.

## Results

### Non-mucoid variants accumulate in cultures of a mucoid *S. aureus* strain

**Figure 1:**
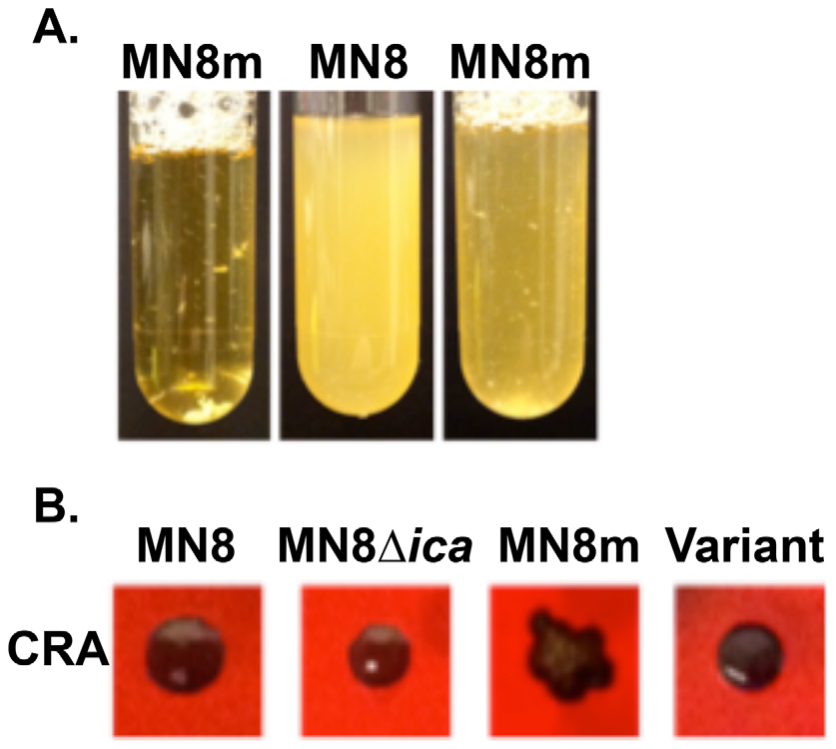
Phenotype characterization of non-mucoid variants isolated from mucoid strain MN8m. A. Liquid cultures in TSBG of highly aggregative mucoid MN8m (tube 1), non-mucoid MN8 (tube 2), and MN8m with mixed appearance (tube 3). B. PNAG production of MN8, MN8Δ*ica*, MN8m, and a non-mucoid variant as indicated by colony morphology on Congo red agar (CRA).

*S. aureus* strain MN8m is a spontaneous PIA/PNAG-overproducing mutant of strain MN8 [15], [20]. A 5 bp deletion in the *icaADBC* promoter region of MN8m is responsible for constitutive *icaADBC* transcription and the constitutive hyper-production of PIA/PNAG that gives the strain its mucoid appearance [15]. It is highly aggregative in liquid culture whereas non-mucoid strains are dispersed, making the culture turbid (Fig. 1A). We found that MN8m cultures frequently exhibited an appearance that was somewhere between that of a turbid, non-mucoid strain and an autoaggregative mucoid strain (Fig. 1A). When these cultures were plated on Congo red agar (CRA), both mucoid colonies, which appear dry with irregular edges, and non-mucoid colonies, which are slick, circular, and occasionally surrounded by a transparent red perimeter, were observed (Fig. 1B).

**Figure 2:**
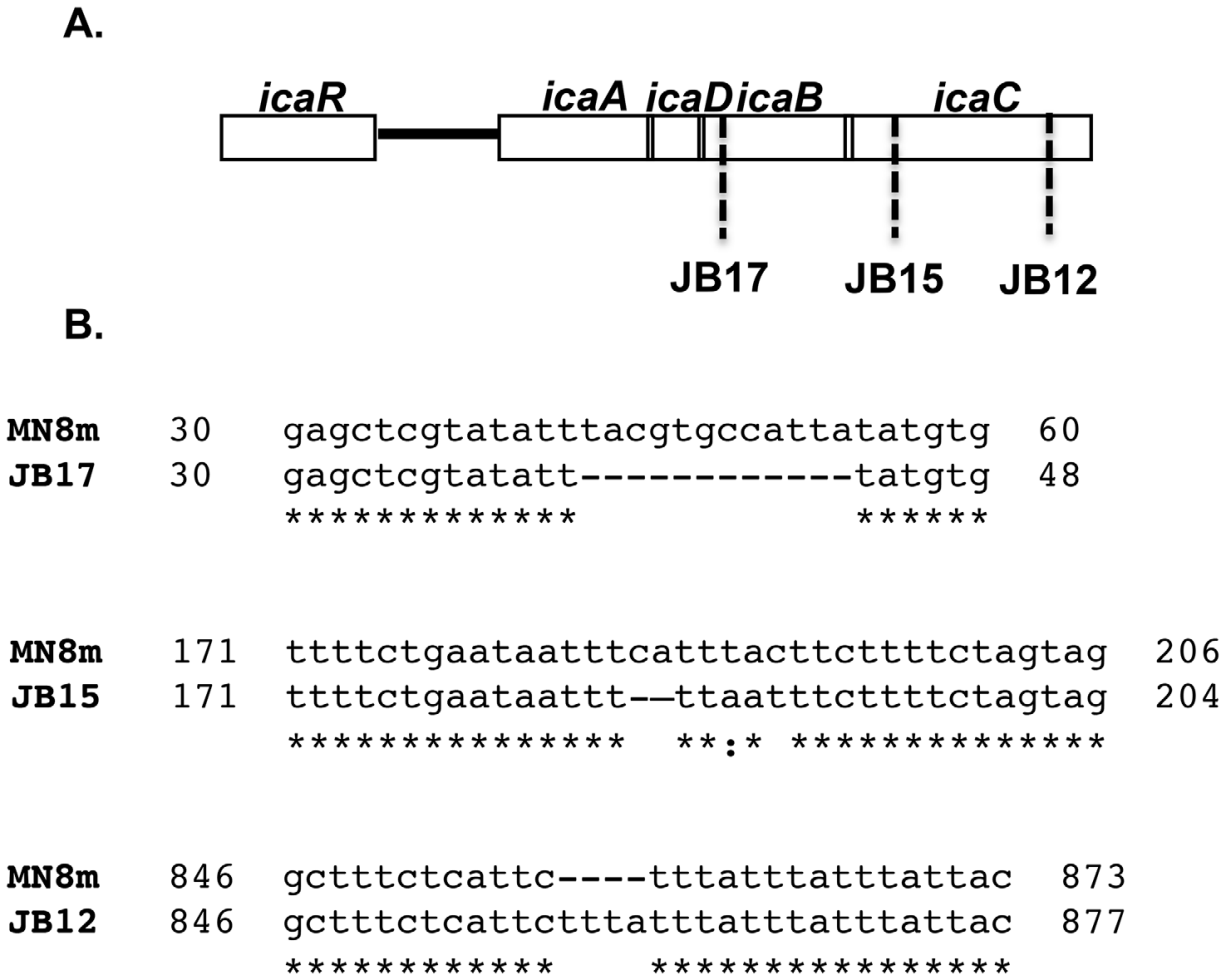
Schematic representation of the *ica* locus of MN8m and non-mucoid variants. A. The location and B. the sequence of mutations detected in non-mucoid variants of MN8m are shown. The position of the 4 bp insertion in the region of repeats identified in *icaC* of the non-mucoid variant JB12 is also shown.

We isolated 15 of the variant colonies and sequenced the *icaADBC* locus (Fig. 2). All of the isolates still exhibited the 5 bp deletion that leads to constitutive *icaADBC* transcription. The sequence of the *icaADBC* locus of four of the isolates was identical to that of MN8m, suggesting a mutation had occurred elsewhere in the chromosome. One of the isolates had a single point mutation within *icaB*. As *icaB* has been shown previously to be dispensable, it is likely that a secondary mutation outside of the *icaADBC* locus was present in this strain as well. One of the isolates had a nonsense mutation at the 5′-end of the *icaC* gene. The remaining nine isolates all shared the loss of a “ttta” tetranucleotide repeat within the *icaC* gene. The insertion led to a shift in the translational reading frame and truncation of the protein; reducing IcaC from 350 amino acids to 303. Because this mutation was the most common amongst the variants, we chose to study it further. We focused on isolate JB12, a tetranucleotide insertion variant.

### The JB12 variant is PIA/PNAG-negative

**Figure 3:**
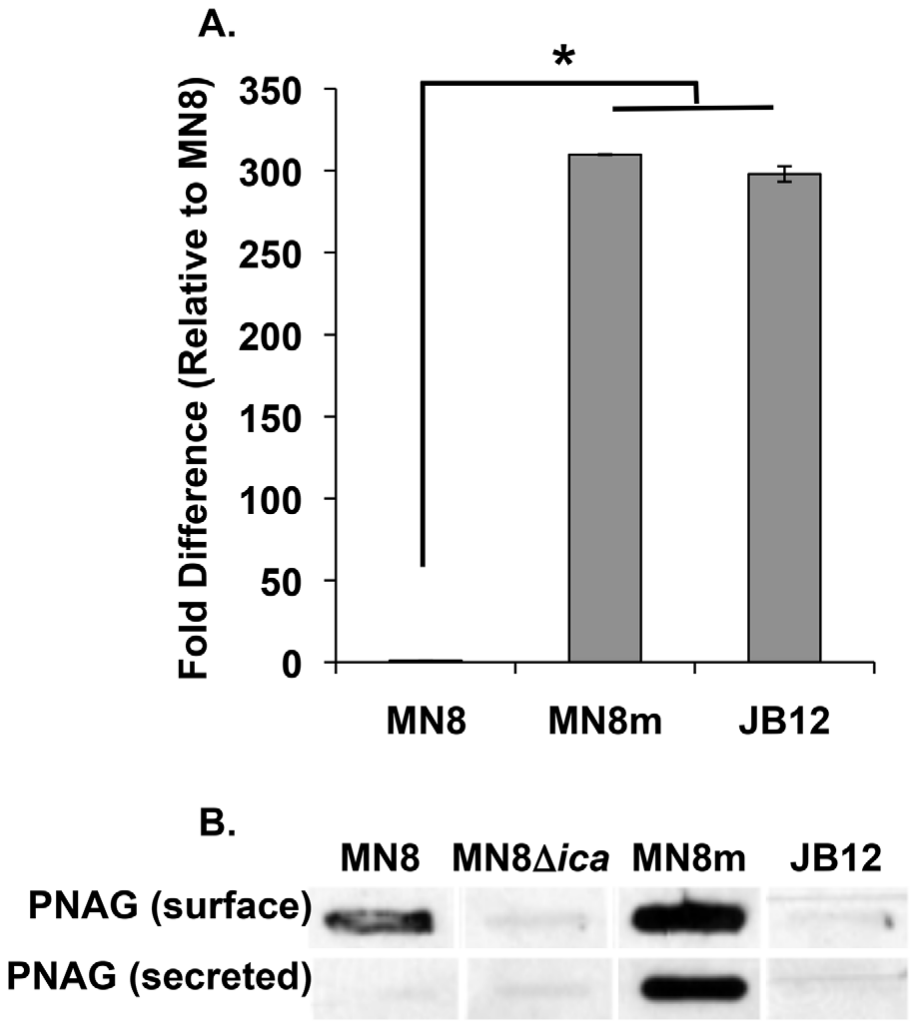
PIA/PNAG production in the non-mucoid variant. A. Real-time RT-PCR analysis of *icaC* mRNA transcript was performed. Bars represent the mean fold difference in transcript amount relative to strain MN8 with n = 3, and error bars indicate the standard deviations. Statistical comparison (unpaired *t* test) of MN8m and JB12 versus MN8 gave a *P* value of <0.001. B. EDTA extractions of surface-associated PIA/PNAG, and spent medium from cultures (secreted polysaccharide) were blotted and probed with PNAG antiserum (MN8m extracts and spent medium were diluted 1∶100).

The *icaADBC* genes are co-transcribed, so to determine levels of the full-length transcript in JB12, we quantified levels of the 3′-most transcript, *icaC*, by realtime RT-PCR. As shown in Fig. 3A, *icaADBC* transcript levels were more than 300-fold greater in MN8m than in the non-mucoid parent strain MN8 and the level remained elevated in the non-mucoid variant. To determine whether or not PIA/PNAG was produced by the JB12 variants, we performed slot-blot analysis using PIA/PNAG-specific rabbit polyclonal antiserum. As depicted in Fig. 3B, no PIA/PNAG was detected on the cell surface or in the spent media of JB12 cultures. Therefore, the mutation in the *icaC* gene resulted in the complete loss of detectable levels of PIA/PNAG.

### Complementation of *icaC* restored PIA/PNAG production

**Figure 4:**
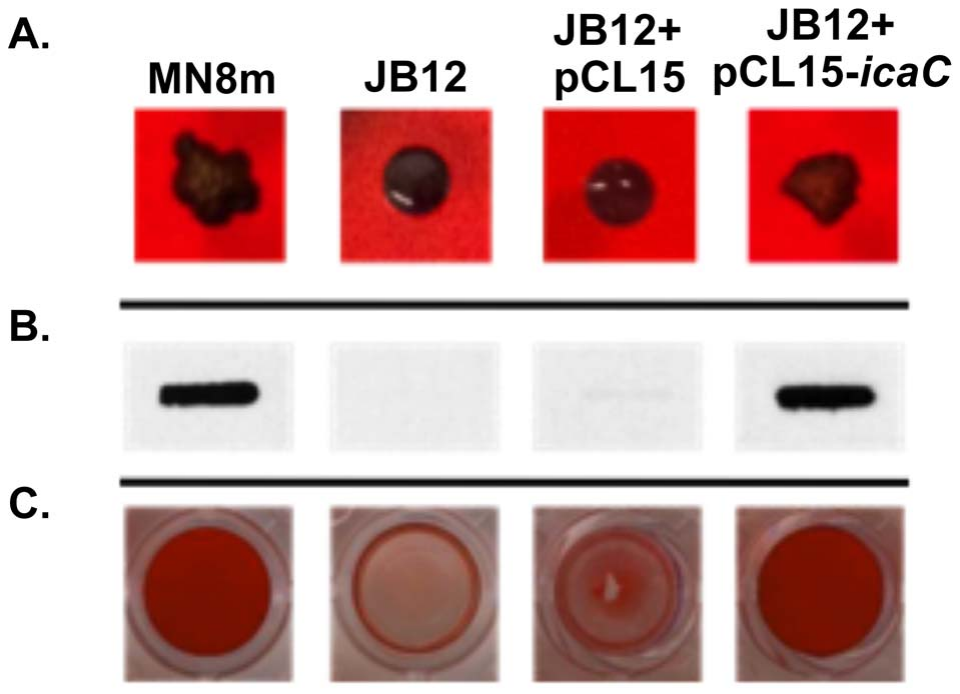
Effect of complementation of JB12 with *icaC* in trans on colony morphology, PNAG production, and biofilm formation. A. Colony morphology of labeled *S. aureus* strains on Congo red agar (CRA) plates. B. PNAG slot blot of cell surface fractions probed with polyclonal anti-PNAG antiserum (MN8m and JB12+pCL15-*icaC* samples were diluted 1∶100 prior to immobilization on membrane whereas JB12 and JB12+pCL15 were not diluted). C. Biofilms formed on microtiter plate wells were stained with safranin and the safranin was quantified by resuspending in acetic acid and measuring OD_562 nm_. Bars represent the mean OD_562 nm_ of 6 replicates, and error bars indicate the standard deviations. Statistical comparison (unpaired *t* test) of JB12+pCL15 (empty vector) versus JB12+pCL15-*icaC* gave a *P* value of <0.001.

To confirm that the nucleotide insertion was responsible for loss of the mucoid phenotype, we complemented the *icaC* gene in trans. Expression of the intact *icaC* gene in strain JB12 from the IPTG-inducible plasmid pCL15, lead to restoration of the mucoid colony morphology on CRA plates (Fig.4A), PIA/PNAG synthesis (Fig. 4B), and biofilm formation (Fig. 4C). Introduction of the empty vector into the variant had no effect.

### The change in tetranucleotide repeat number is reversible

Phase variation is, by definition, a reversible on/off switch. Therefore, if the tetranucleotide repeat insertion was an example of phase variation, then we would expect to isolate variants of JB12 in which the mucoid phenotype was restored. To determine whether or not the phenotype was reversible we plated cultures of JB12 onto CRA. The reversion back to the mucoid phenotype was a rare event and only 1 variant was detected per approximately 45,000 cell divisions. We sequenced 6 mucoid variants from separate JB12 cultures, and all 6 variants sequenced had lost the 4 bp repeat unit that was gained in JB12 meaning that they had reverted back to the MN8m genotype.

To determine whether the mutation in *icaC* was RecA-dependent, we disrupted the *recA* gene in MN8m with the *bursa aurealis* mariner transposon. PIA/PNAG-negative phenotypic revertants were still isolated from the *recA* mutant and of these revertants, the prevalence of the *icaC* repeat mutation was equivalent (6 out of 10 revertants). These results indicate that the mutation was RecA-independent and strongly suggest that the mutation occurred through slipped-strand mispairing.

### Tetranucleotide repeat insertions and deletions occur in clinical isolates

**Figure 5:**
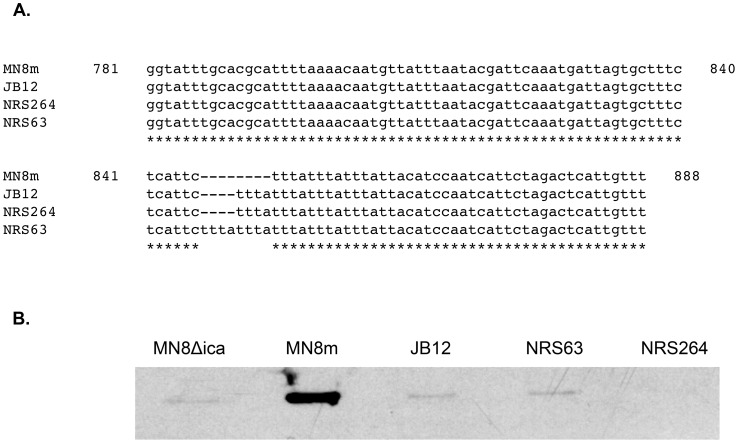
Evidence of *icaC* tandem repeat variation in clinical *S. aureus* isolates, and the effect on PNAG production. A. Nucleotide sequence alignment of the region of repeats identified in *icaC*, comparing mucoid MN8m and non-mucoid isolate JB12 with two published sequences of *S. aureus* clinical isolates, NRS63 and NRS264. B. PNAG slot blot analysis of *S. aureus* strains MN8, MN8Δica, MN8m, JB12, NRS63, and NRS264 probed with PNAG-specific polyclonal antiserum. Due to the very high levels of PNAG produced by MN8m, cell surface extracts from this strain were diluted 1∶100 prior to immobilization on membrane.

To determine whether or not this tetranucleotide repeat indel occurred outside of the laboratory, we examined 51 fully sequenced genomes available in NCBI and 52 sequenced genomes within the NARSA repository. Of these, 9 clinical isolates (∼9%) contained a variation in the tandem repeat region in *icaC* described in this study, demonstrating that the phase variants do occur in vivo (Table 1). Sequences from two representative clinical MRSA strains with deletion or expansion of the repeat units are illustrated in Fig. 5A. The isolates with altered repeat number were PIA/PNAG-negative Fig. 5B.

**Table 1:** Information about strains with variable numbers of tetranucleotide repeats.

Strain designation	Number of tetranucleotide repeats	Culture Source	Other information
NRS264	4	Blood	Isolated from 8 yo male in France. Associated with multiple abscesses and bacteremia.
USA300-ISMMS1	4	No data	
55/2053	4	No data	
TW20 [39]	5	Blood	Representative bacteremia isolate from an outbreak of methicillin resistant *S. aureus* in England
NRS63	5	Blood	Isolated from 50 yo female in Oman. Glycopeptide intermediate resistance.
Bmb9393 [40]	5	Blood	Isolated in Brazil from a bloodstream infection. It was noted that this strain produces *ica*-independent biofilms[40].
Z172 [41]	5	Blood	Isolated from an elderly ICU patient in Taiwan.
T0131 [42]	6	Blood	Multiresistant. Isolated in China.
JKD6008/JKD6009 [43]	6	Blood / wound infection	Isolated in New Zealand. Vancomycin intermediate. The two isolates were from the same patient, both were sequenced, both contained 6 repeats.

We also analyzed a fully sequenced genome using the online server Burrows-Wheeler Tandem Repeat Searcher (BWtrs) to determine whether tetranucleotide repeat indels occur within other genes [21]. Strain MN8m has not been fully sequenced so we chose a strain with an *icaC* tetranucleotide expansion, strain Bmb9393. The genome harbored 59 regions with 3 or more direct tandem tetranucleotide repeats. Out of these 59 regions, 13 exhibited indels when the region was compared to 48 completed *S. aureus* genomes and 438 scaffolds or contigs in the NCBI database. Nine of these indel regions were within intergenic regions, but 4 of them occurred within open reading frames. Two of these indels were in putative phage anti-repressor proteins (SABB_01096 and SABB_02531), one was in a hypothetical phage protein that was not annotated in Bmb9393, and the fourth was in icaC.

### PIA/PNAG over-production is associated with a fitness cost *in vitro*

**Figure 6:**
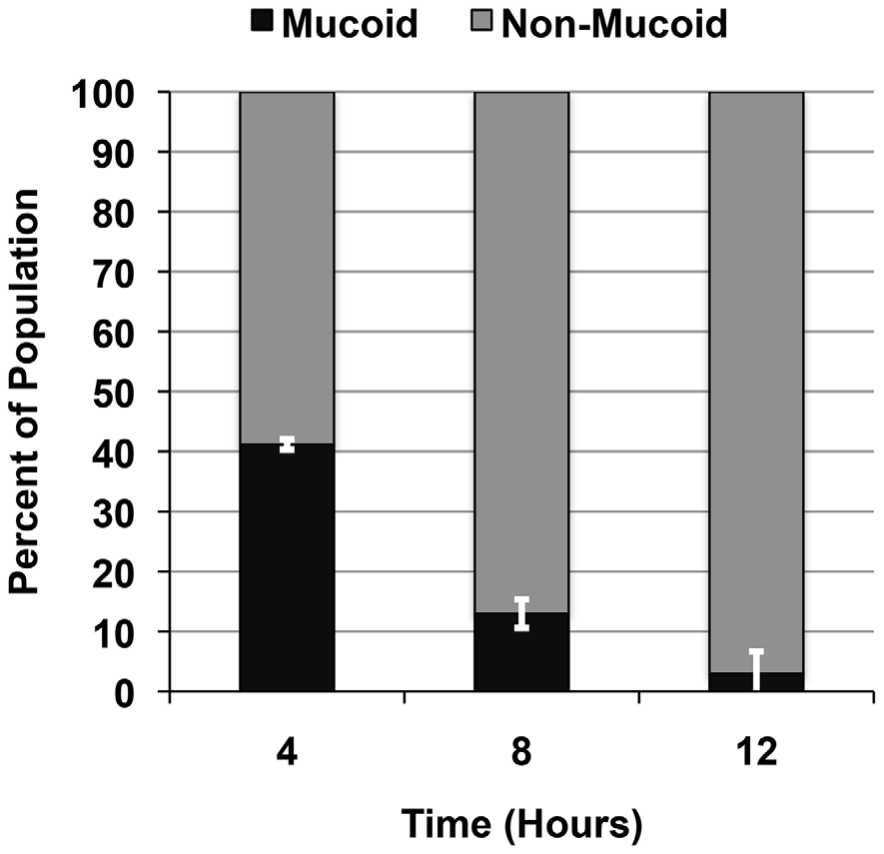
Fitness of MN8m versus JB12. The competitive fitness of MN8m in co-culture with PNAG-negative, non-mucoid JB12 was determined by combining the two strains and enumerating each strain over time. Bars represent the value of each phenotype as percent of total population, determined by enumerating colonies on CRA. Values represent the mean of triplicate samples.

The frequency of reversion from non-mucoid (JB12) to mucoid was very low. We hypothesized that the higher frequency with which nonmucoid variants were isolated from MN8m cultures was due to a fitness cost imparted by high-level PIA/PNAG production. To determine if there was a fitness cost associated with constitutive PIA/PNAG synthesis, we inoculated competitive co-cultures with equivalent numbers of MN8m and JB12 and examined shifts in the population over time by assessing colony morphology on CRA. We observed that there does indeed appear to be significant growth advantage in the PIA/PNAG-negative variant JB12, and that by 12 hours, more than 95% of the culture was non-mucoid (Fig. 6). Direct calculation of the fitness cost of PIA/PNAG over-production versus PIA/PNAG loss resulted in *fit_t_* (relative bacterial fitness) values of +1.401 at 6 hours, and +1.386 at 12 hours, with a value greater than 1 indicating a significant fitness advantage of the JB12 PIA/PNAG-negative phenotype over the mucoid MN8m.

We calculated the generation time for strains with differing levels of PIA/PNAG production. The generation time for MN8, a low-PIA/PNAG-producing strain, was 48 minutes, while that of MN8m, the overproducing strain, was 67 minutes. Interestingly, while the PIA/PNAG-negative strain JB12 had a generation time of 54 minutes, a strain lacking the entire *ica* locus, MN8Δ*icaADBC*::erm had a longer generation time of 59 minutes.

It stood to reason that the difference in generation time was largely responsible for the frequent isolation of non-mucoid variants from MN8m cultures and that the actual frequency of the repeat insertion was low, similar to the rate of repeat deletion in JB12. To minimize the contribution of growth rate, we inoculated liquid medium from single MN8m colonies, plated half of the suspension immediately on CRA to ensure that the starter culture was free from variants, and incubated the culture for only 67 minutes, enough time for a single round of cell division before plating the remainder. We did not detect any variants in 45,000 cell divisions, suggesting that the frequency is less than 1 in 45,000 divisions.

In the absence of a fitness advantage to select for PIA/PNAG-off mutants, we would not expect a high prevalence of mutants in culture. Unlike strain MN8m, strain MN8 produces a more typical, moderate amount of PIA/PNAG. Therefore, to determine whether the *icaC* mutation was only selected for when the parent strain was a PIA/PNAG-overproducer or whether the mutation would occur within an average PIA/PNAG-producing strain, we used high throughput sequencing to determine whether the mutation occurred in MN8. We amplified a 344-bp region of the *icaC* gene that included the tandem repeats and sequenced the product. Out of ∼88,000 reads, ∼140 contained a 4-nt expansion or contraction of the repeat region confirming that the mutation occurs in an average PIA/PNAG-producing strain in vitro.

We also investigated whether the clinical MRSA isolates with *icaC*-off mutations, NRS63 and NRS264, were derived from average PIA/PNAG-producing strains or hyper-producing strains. We performed realtime RT-PCR and found that, unlike strain MN8m, *icaC* transcript levels were moderate and comparable to strain MN8, not strain MN8m (data not shown). We selected 48 colonies with a darker phenotype on CRA from each strain, performed slot-blot analysis using the PIA/PNAG-specific antiserum, and sequenced the *ica* loci. Revertants in which the *icaC* gene reverted to the “on” genotype produced only modest levels of PIA/PNAG (data not shown). Together these data suggest that the “*icaC*-off” strains NRS63 and NRS264 arose from moderate PIA/PNAG producing strains.

### The *ica* locus contributes to survival in the absence of PIA/PNAG

**Figure 7:**
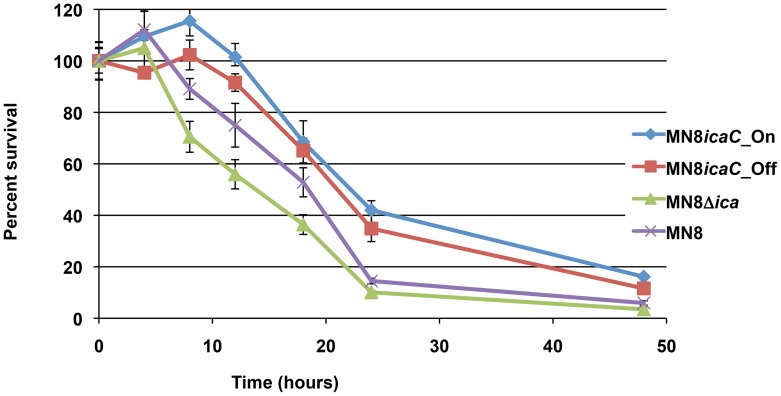
Survival in minimal media. MN8m, JB12, and MN8Δ*ica* were grown in TSBG. The cells were collected and resuspended in minimal media lacking a carbon source to mimic starvation conditions. Points represent the mean percent value of cells enumerated at each time point relative to the starting populations with n = 3, and error bars indicate the standard deviations. Statistical comparison (unpaired *t* test) of MN8m versus JB12 and MN8Δ*ica* versus JB12 at 8 hours and 10 hours gave a *P* value of <0.0001. Comparison of MN8m versus JB12 and MN8*Δica* versus JB12 at 18 hours and 24 hours gave a *P* value of <0.001.

We did not directly measure function to confirm that the IcaA, IcaD, and/or IcaB proteins were functional in JB12; however, complementation of the mucoid phenotype in trans by a copy of the *icaC* gene alone suggests that only the function of IcaC is altered in JB12 and that IcaA, IcaD, and IcaB are still present and functional. We hypothesized that one or more of these proteins may have alternative roles within the cell that contribute to bacterial fitness and that this was the reason why *icaC* was the target for phase variation. Under starvation conditions, when the bacteria were switched to minimal media containing no carbon source, JB12 survived significantly longer than MN8Δ*ica::tet* (data not shown). To confirm that this difference was not due to a secondary mutation introduced during strain passage, three isogenic strains that differed only at the *ica* locus were made. We performed allelic exchange in strain MN8Δ*ica::tet* to replace the tetracycline resistance cassette and the interrupted *ica* locus with the entire MN8m (MN8icaC_On) or the entire JB12 (MN8icaC_Off) *ica* locus on the chromosome. The allelic exchange mutants exhibited survival profiles similar to MN8m and JB12 and both strains survived longer in the minimal media than MN8Δ*ica::tet* (Fig. 7). Production of intact, and presumably functional IcaA, IcaD, and IcaB appeared to significantly increase survival of JB12 under these growth-limiting conditions.

## Discussion

The polysaccharide PIA/PNAG plays an important role in virulence both through its contribution to biofilm formation and immune evasion. In fact, the benefits of PIA/PNAG to survival appear to have resulted in its ubiquitous production by a wide variety of pathogens [22]. It is clear however, that it is not always necessary for survival during infection as PIA/PNAG-negative *S. aureus* and *S. epidermidis* strains have been isolated [23], [24]. In *S. epidermidis*, and in a minority of *S. aureus* strains, PIA/PNAG production can be switched off by the insertion of an IS256 element in the *icaC* gene, however, prior to this study, phase variation of PIA/PNAG expression in *S. aureus* isolates that lack this insertion element was not clear [25], [26].

We noted that it was very difficult to maintain pure mucoid cultures of the PIA/PNAG-overproducing strain MN8m. Over time, MN8m cultures appear to contain a mixture of both mucoid and non-mucoid bacteria. In this study, we investigated the molecular basis for this phenomenon. The most common mutation resulting in the PIA/PNAG-negative phenotype was an expansion of a 4-nt repeat within the *icaC* gene. The rapid increase in the proportion of PIA/PNAG-negative bacteria in these cultures was due to their increased fitness relative to the PIA/PNAG-overproducing parent strain. Of note, we grew our cultures in conical tubes rather than Erlenmeyer flasks, and the depth of the medium likely resulted in microaerobic conditions. The low levels of oxygen during growth may have contributed to PIA/PNAG production and to the relatively low endpoints (OD_600nm_ ∼3.0) in our growth curves [27] and may have also contributed to the growth advantage of the PIA/PNAG-negative mutants. The mutation frequency was very low but the growth advantage allowed non-mucoid variants to take over liquid cultures in time. This resulted in a wide variability in the number of variants present in different cultures depending on the point at which the first variants arose. Therefore, the final number of variants present at the end of the culture period would depend upon the timing of the first mutation event and would be stochastic.

The mucoid strain, MN8m produces approximately 1,000-fold more PIA/PNAG than most clinical isolates. The fitness advantage of the PIA/PNAG-negative variants isolated upon in vitro culture, such as JB12, may be due to the metabolic cost of hyperproduction of PIA/PNAG. During infection, PIA/PNAG plays an important role in immune evasion, and PIA/PNAG-negative variants would likely be more susceptible to neutrophil-mediated killing. Therefore, despite the metabolic cost, selection pressures that favor the PIA/PNAG-negative phase variants are likely less pronounced in vivo. However, skin colonization and ocular infections appear to favor the PIA/PNAG-negative phenotype in *S. epidermidis* and *ica*-negative clinical isolates of *S. aureus* have been detected as well [23], [24], [28]. Furthermore, as PCR amplification of the *ica* genes is often used to demonstrate the capacity to produce PIA/PNAG [29], [30], our finding that a 4-nt indel mutation can shut off PIA/PNAG production suggests that PIA/PNAG negative clinical isolates may be more prevalent than previously appreciated. When we analyzed 103 *S. aureus* genomic sequences, we found that 9 (∼9%) contained a slipped strand mutation in the tandem repeat region in *icaC*. We were able to obtain 2 of these isolates, NRS63 and NRS264, and both were PIA/PNAG-negative. When we isolated “IcaC-on” revertants from NRS63 and NRS264 we found moderate PIA/PNAG production, suggesting that PIA/PNAG hyper-production is not a prerequisite for selection of “IcaC-off” mutants. An immune response against PIA/PNAG could also serve as a selective pressure against PIA/PNAG production. Antibodies against deacetylated PIA/PNAG effectively mediate opsonophagocytosis [31]. Therefore, in the event that the host mounts an effective antibody response, the capacity to switch PIA/PNAG production off could benefit the bacteria in vivo. It could also benefit the bacteria in conditions that favor the planktonic mode of growth.

The 5 bp deletion in the MN8m *ica* promoter leads to constitutive *icaADBC* transcription and the accumulation of at least 300-fold more transcript than the clinical isolate parent strain MN8. Such high-level transcript production and protein synthesis would seem to be metabolically costly even in the absence of PIA/PNAG production. We therefore found it somewhat surprising that the most common mutation (9 out of 15 variants) occurred within the last gene in the operon (*icaC*) and that *icaADBC* transcript was still being produced at MN8m levels. Furthermore, the insertion sequence IS256 has been shown to turn PIA/PNAG synthesis off through insertional inactivation of *icaC* [26] suggesting again that mutation of *icaC* is the preferred “off switch” for PIA/PNAG production. We tested our hypothesis that there might be some advantage to continuing synthesis of IcaA, IcaD, and IcaB even though PIA/PNAG is not produced in the absence of IcaC. Our results indicate that under nutrient-limiting conditions, possession of functional *icaADB* genes was advantageous for survival. The basis for this survival advantage is unclear at this time. Further work is necessary to conclusively determine whether or not the IcaA, IcaD, and IcaB proteins function in some capacity in addition to PIA/PNAG synthesis.

In conclusion, we found that the RecA-independent expansion or contraction of a 4-nt tandem “ttta” repeat shifts the reading frame of *icaC*, leading to a premature stop codon, truncating the protein at 303 amino acids; 47 amino acids shorter than full-length protein. Structural prediction indicates that the mutation disrupts a transmembrane domain of IcaC, and we found that the mutation resulted in the complete abrogation of PIA/PNAG production. We found that the mutation frequency was low, but that in vitro, elevated production of PIA/PNAG carried a fitness cost and consequently, PIA/PNAG-negative phase variants quickly increased in number relative to PIA/PNAG over-expressers. Of note, IcaC appears to be the chosen target for phase variation in PIA/PNAG production and loss of this protein appears to confer a survival advantage under nutrient-poor conditions relative to loss of the entire operon suggesting that the other proteins encoded within the *ica* locus could have some other function. Alternatively, PIA/PNAG precursors could accumulate within the bacterial cells in the absence of IcaC, and affect growth. Studies to determine the effect of the IcaADB proteins on growth are underway.

## Materials and Methods

### Staphylococcal strains and media

*Staphylococcus aureus* strain MN8 is a clinical isolate from a case of toxic shock syndrome [32]. Strain MN8m was a spontaneous mutant isolated from a chemostat culture of strain MN8 [20]. Strain SA113Δ*ica* was provided by Dr. Sarah Cramton [12] and the mutation was transduced to strain MN8 by phage 80α to produce MN8Δ*ica::tet*. Strain MN8Δ*ica::tet* was complemented by allelic exchange with the *ica* loci from the entire MN8m and JB12 strains to produce strains MN8icaC_On and MN8icaC_Off, respectively. To this end, the *ica* loci were amplified by primers SA11 and SA12 as previously described [12] cloned into the pMAD vector, and allelic exchange was performed as previously described [33]. Mutants were tetracycline sensitive and allelic exchange was confirmed by sequencing the *ica* loci. A *recA* mutant of strain MN8m was produced by transducing the *bursa aurealis* transposon from strain NE805 (NARSA repository) using phage 80α. NRS264 and NRS63 are sequenced clinical isolates obtained from the NARSA repository. NRS264 was associated with bacteremia and abscess and NRS63 was a bacteremia isolate. All strains were grown aerobically at 37°C on tryptic soy agar (TSA) plates containing the appropriate antibiotic. Liquid cultures were in tryptic soy broth containing 1% glucose (TSBG), incubated in air at 37°C, 200 rpm in 5 mL in 50 mL conical tubes (microaerobic conditions). Congo red agar was composed of brain heart infusion (BHI) agar +3.6% sucrose +0.5% glucose +0.08% congo red.

### Plasmids, primers, and cloning

Plasmid purifications were performed using the QIAprep Spin Miniprep kit (Qiagen, Valencia, CA) according to the manufacturer's instructions. Primers were custom synthesized by Integrated DNA Technologies (Coralville, IA). Restriction enzymes were purchased from New England Biolabs (Beverly, MA). The *icaC* gene was cloned into the isopropyl-β-d-thiogalactopyranoside (IPTG)-inducible vector pCL15 (kindly provided by Dr Chia Lee, University of Arkansas) [34]. The gene was PCR amplified from strain MN8m genomic DNA with the primer set, icaCSphIFwd (5′-CCGCGCATGCCAAAAATGGCAGAGAGGAAGA-3′) and icaCKpnIRev (5′-CCGCGGTACCCCGCGTGTTTTTAACATAGC-3′). Initial cloning was performed in *E. coli* using the pCR4TOPO vector (Invitrogen, Grand Island, NY) according to manufacturer's instructions. The genes were digested from the cloning vector with the appropriate restriction enzymes, purified after gel electrophoresis using the QIAquick Gel Extraction kit, and ligated into pCL15 using Ready-To-Go T4 DNA ligase (GE Healthcare, Piscataway, NJ). After passage through *E. coli*, all plasmid constructs were transformed into the restriction-deficient *S. aureus* strain RN4220 according to the method of Lee [35]. Constructs were transferred to other strains of *S. aureus* by transduction with phage 80α.

### Growth/fitness determination

To generate a bacterial growth curve for use in calculating strain generation time, TSBG was inoculated with individual bacterial colonies and gently sonicated for 30 seconds to break apart clusters. Each culture was diluted to OD_600nm_ of 0.1 and used to inoculate fresh TSBG 1∶100, with separate tubes for each time point. The cultures were incubated at 37°C, 200 rpm. At each time point the cultures were gently sonicated, the OD_600 nm_ measured, and plated on CRA plates to monitor population mucoid/non-mucoid phenotype over time and ensure that variants did not arise to influence observed growth rates. Logarithmic growth was determined to occur between 180 and 360 minutes for each of the strains. For this time period, the A_600_ measurements were converted into log_2_ values, and the generation time was calculated as the inverse of the slope of the line of best fit.

For the competitive fitness assay, cultures were grown overnight in TSBG, and gently sonicated for 30 seconds. Each culture was diluted to a concentration of 10^3^ cells and mixed 1∶1 with MN8m + JB12, with separate tubes for each time point. The cultures were incubated at 37°C, 200 rpm. At each time point the cultures were gently sonicated, serially diluted and plated in triplicate on CRA plates for CFU counting. Calculation of the difference in fitness was determined using the function derived from Sander *et al.* [36] LN(((nm_t_/m_t_)/(nm_t-1_/m_t-1_))∧(1/gen)) where nm*_t_* and m*_t_* represent the non-mucoid and mucoid cells, respectively at a given time *t*. While nm_t-1_ and m_t-1_ denote the quantity of non-mucoid and mucoid cells at the preceding timepoint. The quotient of the ratios was standardized with the exponent 1/generation, with the assumption that cell numbers determined at 24 hours represents approximately 17 generations. The relative bacterial fitness for a given time was calculated as *fit_t_*  = 1+S*_t_*. The fitness value is equal to 1 if there is no difference in fitness between the competing strains, less than 1 if the non-mucoid phenotype reduces fitness, or greater than 1 if the non-mucoid phenotype increases bacterial fitness.

### Biofilm assay

Microtiter plate assays for biofilm formation were performed essentially as described previously by Christensen *et al*. [37] with minor modifications. Cultures were grown overnight in 4 ml of TSBG or TSBG +10 µg ml^−1^ chloramphenicol, diluted 1∶200 in the same media or media with 1mM IPTG added for plasmid induction, and aliquoted into 96-well polystyrene flat-bottom microtiter plates (Greiner Bio-One, Monroe, N. Carolina). After 24 hours at 37°C, the wells were emptied and washed once with phosphate-buffered saline (PBS). The plates were dried at room temperature, stained with 200 µl safranin for 1 minute, washed gently with water, and allowed to dry. The biofilms were assessed qualitatively by visual inspection and images were taken using a digital scanner. The safranin was then resuspended in 200 µl 33% acetic acid and the wells were analyzed by spectrophotometry at OD_562 nm_ using a 96-well plate spectrophotometer.

### Quantitative real-time RT-PCR

RNA was isolated from exponentially growing bacteria, following induction with 1 mM IPTG for 2 hours if pCL15 was present, using the Qiagen RNeasy kit (Qiagen, Valencia, Calif.) according to the manufacturer's instructions. Contaminating DNA was digested with Turbo DNAse (Ambion, Austin, Texas), and the mRNA transcript levels were measured by quantitative reverse transcriptase (RT)-PCR. Reverse transcription of 1 µg of RNA was performed using the Tetro cDNA synthesis kit (Bioline, Taunton, Mass.) according to manufacturer's instruction, and 10 pmol icaCRTRev (5′-CGTTCCAATAGTCTCCATTTGC-3′), and 16SRTRev (5′- TATGCATCGTTGCCTTGGTA-3′). Controls for DNA contamination contained no reverse transcriptase. SensiMix SYBR & Fluorescein mix (Bioline, Taunton, Mass.) was used for the quantitative real-time PCR with the primer sets: icaCRTFwd (5′-CGAACAACACAGCGTTTCAC-3′) and icaCRTRev, or 16SRTFwd (5′-GAACCGCATGGTTCAAAAGT-3′) and 16SRTRev.

### PIA/PNAG slot blot analysis

PIA/PNAG slot blots were performed essentially as described previously by Cramton et al. [27] with minor modifications. Bacteria were grown overnight in TSBG or TSBG +10 µg ml^−1^ chloramphenicol +1 mM IPTG. For cell surface extracts 10^9^ cells were collected by centrifugation, washed once with PBS, and resuspended in 250 µl of 0.5 M ethylenediaminetetraacetic acid (EDTA). To analyze secreted PIA/PNAG, 250 µl of spent media was retained. All samples were incubated in boiling water for 5 minutes, cooled, and incubated at 65°C for 1 hour with 20 µl proteinase K. Samples were boiled for an additional 5 minutes to inactivate the protease, diluted in PBS, and immobilized on nitrocellulose with a vacuum manifold. Blots were blocked overnight at 4°C in 5% bovine serum albumin, probed with 1∶5,000-diluted rabbit antiserum specific for PIA/PNAG (kindly provided Dr. Gerald B. Pier) [7] for 2 hours at 21°C, washed, and probed with 1∶10,000-diluted goat anti-rabbit immunoglobulin-horseradish peroxidase conjugate for 1 hour at 21°C. Bands were visualized with the ECL Plus western blotting detection system (GE Healthcare).

### High-throughput sequencing of the repeat region

MN8 was cultured for 4 hr at 37°C. DNA was purified using the DNeasy Blood and Tissue kit (Qiagen) and amplified by PCR using SSSeqFWD (5′-TCGTCGGCAGCGTCAGATGTGTATAAGAGACAGGGAACGTTACCAGCTTTTCATATTC-3′) and SSSeqREV (5′-GTCTCGTGGGCTCGGAGATGTGTATAAGAGACAGCCACCGCGTGTTTTTAACATAGC-3′). The PCR product was sequenced at the Nucleic Acids Research Facilities at VCU using Illumina MiSeq. Sequencing yielded ∼88,000 paired end reads, which were compared to the MN8 parent sequence using JBrowse to detect indels [38].

### Survival in nutrient-limited conditions

Liquid cultures were initially grown shaking aerobically in TSBG for 24 hours. Bacteria were collected by centrifugation, and equal numbers of bacteria for each sample were resuspended in equal volumes of MOPS minimal media (Teknova, Hollister, Ca) with no glucose, or other carbon source. The cultures were incubated at 37°C while shaking. At each time point the cultures were serially diluted and plated in triplicate on TSA plates for CFU enumeration. Each strain was analyzed in technical triplicate and biologic replicates.
